# Moyamoya Disease with Non-Functional Pituitary Macroadenoma: A Case Report of a Rare Presentation

**DOI:** 10.7759/cureus.56728

**Published:** 2024-03-22

**Authors:** Rizwan Ullah, Jubran Al Balushi, Nadia Nishat, Hafiz Muhammad Faizan Mughal, Gayatri Misra, Shehriyar

**Affiliations:** 1 Internal Medicine, Hayatabad Medical Complex, Peshawar, PAK; 2 Medicine, University College Dublin, Dublin, IRL; 3 Family Medicine, Adichunchanagiri Institute of Medical Sciences, Mandya, IND; 4 Internal Medicine, Allama Iqbal Memorial Teaching Hospital, Sialkot, PAK; 5 Medicine, American University of Antigua, Osbourn, USA

**Keywords:** carotid arteries stenosis, collateral circulation, cerebral artery stenosis, intracerebral haemorrhage, revascularization procedures, cerebral angiography, ischemic stroke, hemorrhagic stroke, pituitary macroadenoma, moyamoya disease

## Abstract

Moyamoya disease (MMD) is a rare neurological condition characterized by brain blood vessel narrowing, leading to collateral vessel formation. Diagnosis typically involves cerebral angiography and magnetic resonance angiography (MRA), with surgical revascularization often providing superior outcomes.

Here, we present the case of a 55-year-old woman with hypertension, diabetes, and a history of ischemic stroke. She recently experienced a hemorrhagic stroke due to MMD, compounded by a non-functional pituitary macroadenoma.

Recognizing signs of a hemorrhagic stroke is crucial to prevent future occurrences and ensure optimal outcomes. However, our understanding of the connection between MMD and pituitary macroadenoma remains incomplete. Further research is essential to refine diagnostic techniques and treatment strategies. Through continued research and awareness, we can strive for improved outcomes and an enhanced quality of life for individuals affected by MMD and its complications.

## Introduction

Moyamoya disease (MMD) was first documented in Japan in 1957 but has since been observed in other Asian regions. The term "moyamoya," derived from Japanese, refers to the distinctive angiographic appearance of collateral circulation in affected individuals' brains. This progressive condition primarily narrows the distal internal carotid arteries on both sides, as well as the anterior cerebral arteries (ACA) and middle cerebral arteries (MCA), leading to collateral vessel formation [1].

The disease, rare with an estimated incidence of 0.086 cases per 100,000 people, is more prevalent in females [2,3]. In some cases, it has been associated with a pituitary macroadenoma. For example, studies have documented MMD accompanied by prolactin-producing pituitary adenomas, suggesting a potential connection between the two conditions [4]. Additionally, cases involving growth hormone-secreting pituitary adenomas alongside MMD propose an association between excess growth hormone and vascular disease progression [5,6]. A case of unilateral MMD with an intracranial aneurysm and a non-functional pituitary adenoma underscores the importance of evaluating MMD in individuals experiencing acute cerebrovascular changes [7]. Collectively, these studies suggest a potential relationship between MMD and pituitary macroadenoma, although further research is required to fully elucidate the underlying mechanisms.

Now, we present the case of a 55-year-old woman with a medical history of hypertension, diabetes mellitus, and a prior ischemic stroke who experienced a hemorrhagic stroke. Further investigation confirmed the diagnosis of MMD in association with a non-functional pituitary macroadenoma.

## Case presentation

Upon admission to our tertiary care hospital, a 55-year-old woman from Peshawar, Pakistan, with a medical history of hypertension, diabetes mellitus, and a prior ischemic cerebrovascular accident (CVA) one year ago, presented with acute symptoms. These included a severe headache, vomiting, right-sided weakness, and aphasia, ultimately resulting in a deep coma. Notably, she had no known history of head trauma or familial predisposition to such conditions.

Her vital signs were recorded as follows: blood pressure (BP) 210/140 mmHg, pulse rate (PR) 84 bpm, oxygen saturation 93%, temperature 98°F, and random blood sugar (RBS) level 235 mg/dl. A neurological examination revealed a Glasgow Coma Scale (GCS) score of 3/15 (normal 15/15), NIH stroke scale on arrival was 3. While bilateral pupils were equal and reactive, there were absent deep tendon reflexes on the right side, with motor strength rated as 0/5 on that side and 5/5 on the left. The right-sided plantar reflex was also muted, and there was no evidence of neck stiffness.

**Figure 1:**
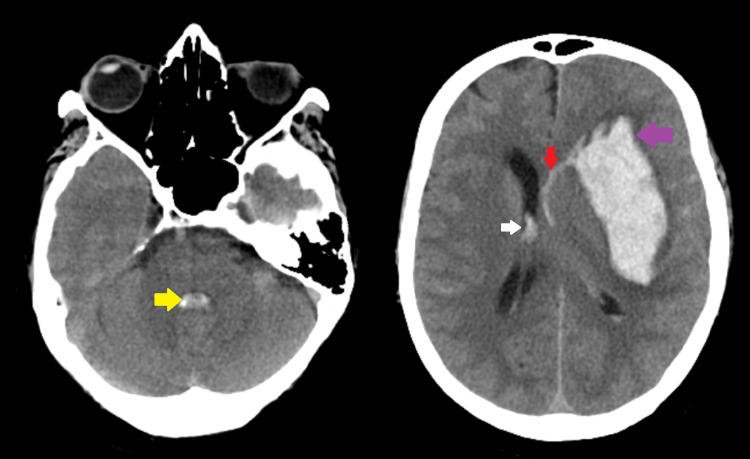
CT brain without contrast reveals subarachnoid hemorrhage (red arrow), intraparenchymal hemorrhage in the left frontotemporal lobe and lentiform nucleus (purple arrow), intraventricular extension (white arrow), and brainstem bleed (yellow arrow)

Subsequent examinations did not reveal any notable findings. Laboratory tests indicated a slightly elevated white blood cell count. CT brain imaging showed intraparenchymal hemorrhage in the left frontotemporal lobe and lentiform nucleus, with intraventricular extension and brainstem bleeding. The ventricular size remained normal despite the hemorrhage, with a mild midline shift noted. Furthermore, subarachnoid hemorrhage was observed in the left cerebral hemisphere, accompanied by cerebral edema (Figure 1).

After consulting with neurosurgery, the patient was admitted for blood pressure optimization. She was treated with a mannitol infusion (1 g/kg loading dose followed by 0.3 g/kg every eight hours for three days), oral nimodipine at 60 mg every 4 hours for 21 days, and intravenous dexamethasone at 8 mg followed by 4 mg twice daily for three days. Her systolic blood pressure was maintained at around 160 mmHg, and prophylactic intravenous ceftriaxone was administered at 2g twice daily.

**Figure 2:**
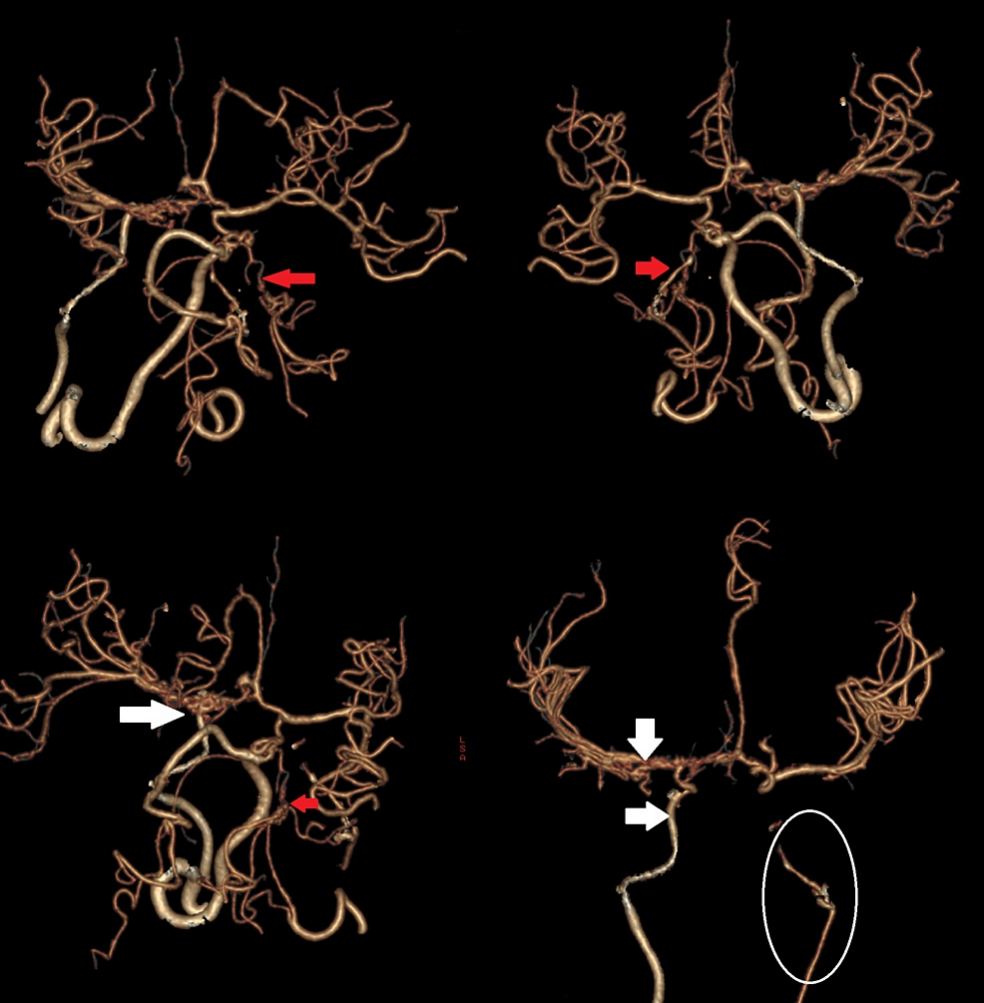
CT angiography (CTA) of the brain reveals stenosis in the cervical, carotid canal, and cavernous part of the right internal carotid artery (white circle), a narrowed left internal carotid artery with collateral vessels in the regions of left M1 and M2 (white arrows), and the right posterior cerebral artery (red arrows)

By the third day, the patient's Glasgow Coma Scale (GCS) score had improved to 9/15. CT angiography (CTA) of the brain revealed stenosis in the cervical, carotid canal, and cavernous part of the right internal carotid artery, as well as a narrowed left internal carotid artery and collateral vessels in the regions of the left M1 and M2 and the right posterior cerebral artery (Figure 2).

**Figure 3:**
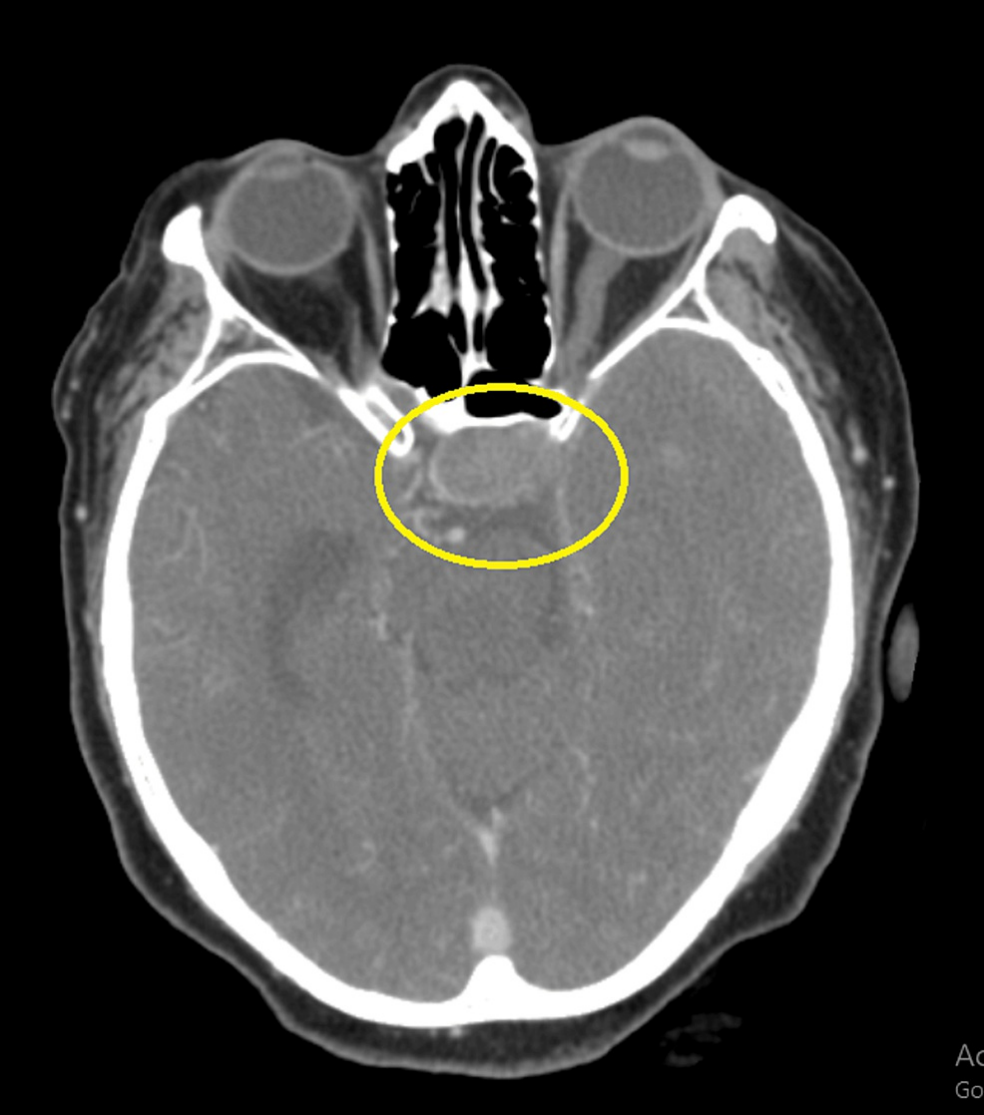
A contrast-enhancing soft tissue lesion measuring approximately 1.6 x 2.8 x 8 cm in the pituitary gland region suggests a pituitary macroadenoma bulging into the left cavernous sinus (yellow circle)

Furthermore, an observed enhancement of the soft tissue lesion measuring 1.6 x 2.8 x 8 cm was observed in the pituitary gland region. This lesion represents a pituitary macroadenoma bulging into the left cavernous sinus (Figure 3).

Following normal hormonal profile assessments for pituitary adenoma, which included follicle-stimulating hormone (FSH), luteinizing hormone (LH), thyroid-stimulating hormone (TSH), and growth hormone (GH), the patient was referred to the neurosurgery department for further management. Although the patient presented with hemorrhagic stroke, which can be due to hypertension, there is also an association between MMD and renovascular hypertension (discussed below). Imaging findings are suggestive of Moyamoya disease, concomitant with a non-functional pituitary adenoma.

## Discussion

MMD, characterized by progressive constriction of brain blood vessels, notably affects the terminal internal carotid arteries bilaterally, along with the anterior and middle cerebral arteries [1]. Our patient's CTA brain scan revealed bilateral involvement of the carotid arteries alongside the left middle cerebral and right posterior cerebellar arteries. Notably, females are predominantly affected by this condition [3].

Several studies have explored the link between MMD and pituitary macroadenoma. For instance, two cases reported prolactin-producing pituitary adenomas, suggesting a potential correlation [4]. Similarly, two other cases indicated an association between growth hormone-secreting pituitary adenomas and MMD [5,6]. Additionally, a case involving unilateral MMD with an intracranial aneurysm and a non-functional pituitary adenoma underscores the importance of assessing acute cerebrovascular changes [7]. In our case, CTA findings suggested MMD and a non-functional pituitary macroadenoma. However, further research is warranted to fully elucidate the underlying mechanisms.

Typically, MMD presents as an ischemic event. A study conducted on 528 patients in China revealed that most cases presented with ischemia (63%), with hemorrhagic (37%) presentations more prevalent among adults (58%). The ischemic type generally has a favorable prognosis and lower re-bleeding chances [8].

Recent studies have highlighted the occurrence of vascular narrowing in both intracranial and extracranial vessels, particularly in the renal artery, among patients with Moyamoya disease. Renovascular hypertension (RVH) is recognized as a significant contributor to hypertension in these patients. However, the underlying mechanisms linking moyamoya disease and RVH remain poorly understood, and there is no consensus on the optimal treatment approach [9].

The Japanese research committee established diagnostic criteria for MMD, including occlusion at the terminal internal carotid artery (ICA) or proximal anterior cerebral artery/middle cerebral artery (ACA/MCA), presence of collateral vessels, and bilateral vessel involvement [10]. Revised criteria in 2012 incorporated findings from magnetic resonance angiography (MRA); patients exhibiting unilaterally affected vessels were classified as probable Moyamoya disease. Cerebral angiography stands as the gold standard investigation method [11].

In cases of hemorrhagic presentation, it is imperative to discontinue antiplatelet and anticoagulation medications promptly, consider blood pressure control, and evaluate for surgical interventions. While aspirin is recommended for secondary stroke prevention, caution is advised for long-term use due to the risk of intracerebral hemorrhage. Clopidogrel serves as an alternative. Surgical interventions significantly reduce the likelihood of stroke recurrence [12]. Consequently, we referred our case to neurosurgery for further intervention.

## Conclusions

Moyamoya disease, a rare condition, results in arterial narrowing and collateral vessel formation in the brain's blood vessels. Our case suggests a potential link between MMD and pituitary macroadenoma, prompting further investigation. Accurate cerebral and magnetic resonance angiography diagnosis is crucial, guided by typical imaging findings. Prompt management of hemorrhagic presentation entails discontinuing antiplatelet and anticoagulation medications and considering antihypertensive therapy. Surgical revascularization reduces stroke recurrence compared to conservative approaches. This underscores the necessity for comprehensive evaluation and multidisciplinary management involving neurology, neurosurgery, and endocrinology specialties to ensure optimal care.
